# Efficient power macromodeling approach for heterogeneously stacked 3d ICs using Bio-geography based optimization

**DOI:** 10.1371/journal.pone.0264181

**Published:** 2022-02-22

**Authors:** Faisal Siddiq, Yaseer Arafat Durrani

**Affiliations:** Faculty of Electronics and Electrical Engineering, University of Engineering and Technology, Taxila, Punjab, Pakistan; National Institute of Technology Silchar, India, INDIA

## Abstract

Low-power consumption has been always a crucial design constraint for an efficient intellectual property based three-dimensional multi-core system that cannot be ignored easily. As the complexity increases due to the number of cores/stacks/ layers in 3D digital systems, the challenges to handle power can be more difficult at a high abstraction level. Therefore, the low-power approach gives designers an opportunity to estimate and optimize the power consumption in the early stages of design phases. The accurate power estimation through the macro-modeling approach at high-level reduces the risk of redesign cycle and turn-around time. In this research, we have presented an improved statistical macro-modeling approach that estimates power through statistical characteristics of randomly generated input patterns by using Biogeography Based Optimization. These input patterns propagate signals into an IP-based 3D digital test system. In experiments, the test system is based on four 8 to 32- bits heterogeneous cores. The response of the power is monitored by applying the well-known Monte Carlo Simulation technique. The entire power estimation method is performed in two major steps. First, the average power is estimated for an IP-based individual core. Second, the average power for bus-based Through-Silicon-Via is estimated. Finally, the cores and B-TSVs are integrated together to construct a 3D system. Then the average power for complete test systems is estimated. The experimental results of the statistical power macro-model are compared with the commercial Electronic Design Automation power simulator at the operating frequency of 100 MHz. The average percentage error of the test system is calculated as 8.65%. For the validation of these results, the statistical error analysis is additionally performed and reveals that our proposed macro-model is accurate in terms of percentage of error with a feasible amount of time.

## 1 Introduction

In Very Large Scale Integration (VLSI) design, low-power, performance, and area is a major concern for designers. It is difficult for designers to maintain the balance between these performance parameters and provide an optimal solution. But, low-power consumption has been always a crucial design constraint for an efficient intellectual property (IP)-based three-dimensional (3D) multi-core system that cannot be ignored easily. The main reasons are:

1. Nano size of transistor and high operating frequencies increased the cooling cost
2. Modern portable applications have a battery-life constraint

Therefore, a low-power approach that gives designers an opportunity to estimate and optimize the power consumption in the early stages of design phases. The high-power consumption not only creates challenges for power sources but also has significant effects on portability, reliability, and cost of the device. The low-power includes all efforts to optimize power constraints while meeting design metrics. It is further divided into two sub-categories: Power estimation and optimization. Power estimation techniques are used for the power estimate of digital circuits. It is desirable to develop a power estimation technique at a higher level of abstraction. The demand for low-power design has motivated researchers in the area of power estimation and optimization. These techniques are applied at almost all levels of abstraction. Low-level estimation techniques are used at the later design stage and are less used due to their complexity and high time consumption. On the other hand, high-level estimation techniques give an early and accurate estimate of power and budget, with reduced turn-around time.

Conventional design approaches are not adequate to deal with power estimation and optimization of modern multi-core devices. It is difficult to maintain the balance between speed and efficiency. Therefore it is a challenging task to approach a low-power successful 3D Integrated Circuit (IC) design.

In the initial phase of designing, Electronic Design Automation (EDA) tools help designers with power estimation, analysis and optimization. They improve the performance parameters of 3D IC design. It also allows the power management of budget during the initial design phases [1].

As system complexity increases for today’s modern 3D digital systems, verification of the system is a very time-consuming procedure. The design time and cost of the digital system can be minimized by re-using the pre-designed blocks. IP blocks are one of the solutions for the problems that are associated with multi-core systems. Designers are at ease by using pre-validated components and IPs in system design. Re-use of IP is just like plug-and-play and also includes interconnect buses and hierarchical infrastructures. In the initial design phase, designers are very much interested and keen on interconnect organization, libraries, memory, and IP power requirements. It also reduces the evaluation and turnaround time to the market. Due to the recent time-to-market challenges, designers must estimate the power and area accurately and rapidly for complex and diverse applications.

In light of the above-mentioned challenges, power estimation models are used at all abstraction levels with different power and speed requirements. Among various approaches, the accurate power estimation through the *macro-modeling* approach at high-level reduces the risk of redesign cycle and turn-around time. Application of power macro-modeling on different IP blocks and multiple cores of a 3D digital system; requires statistical knowledge of input patterns.

Therefore power macro-modeling of 3D ICs is a useful technique for both front and back-end designers to predict the power, thermal and area requirements of the overall system. The earlier power analysis of 3D ICs manages the power requirements during the initial system design phase. An improved and efficient power macro-modeling for IP-based heterogeneous 3D digital system is proposed by using a new statistical variable Signal Variance (SV). A new statistical model for bus-based through-silicon-via (B-TSV) is presented. An optimized input patterns are generated by using Bio-geography based optimization algorithm and it also covers full span of input patterns. Moreover, there is no precedent study on the adaption of BBO for input pattern generation for power estimation of any 3D digital system. Therefore we expliot this option of BBO and used for optmized input patterns. The effectiveness of BBO will be evident from the experimental results. A detailed statistical analysis is given for the validity of the proposed model.

Section-II describes the literature review, problem formulation of 3D IC design. Section III describes the power macro-modeling and analysis of different components of 3D ICs. Section IV discusses and shows the experimental results. Finally, Section V gives summary, conclusion and future directions of the research work.

## 2 Literature review

Due to the scaling of technology, the number of transistors is increased in the same area. It also affects the cooling effects and packaging of a chip. Therefore early power estimation is necessary for design space exploration at the RTL level [2]. For efficient management of power in FPGA-based designs, accurate power estimation is required. It is hard to find the input patterns/variables that affect power consumption. A simulator is proposed in [3] for power estimation depending upon input data. By the early and accurate estimation of multiplier, blocks can help designers for the efficient designing of low-power applications [4]. An FPGA based model generates the SPICE netlist depending upon the architecture and also enables the precise power estimation of architecture [5].

A linear power model that estimates the power of FPGA-based simulators of a microprocessor is proposed in [6]. It predicts cycle-wise power and is comparable with a commercial gate-level power estimation tools. A model is presented in [7] for dynamic power estimation for specific applications implemented on FPGAs. A profiling-based neural network model is used for dynamic power estimation. Different numbers of inputs are feed as category-wise instructions targeted for specific FPGA applications. Due to various factors, there is heterogeneity in the dynamic power of chips for Fin-FET-based microprocessors [8]. A high-level area and power estimator, based on analytical modeling is proposed in [9]. It helps designers to have an early estimate of the power and area of FPGA-based accelerators. A fast and accurate power model for full-custom hardware embedded in heterogeneous systems at a high-level of abstraction is presented in [10]. Hardware basic building blocks are considered at the RTL level in the characterization phase of power estimation. A power macro-modeling technique based on statistical input characteristics for DSP architectures is presented in [11]. As power is a function of its primary inputs, therefore statistical inputs are generated by using the evolutionary algorithm. It also considers the Spatio-temporal correlation between the different input patterns. A hybrid system-level model for MPSoC is presented in [12].

Power estimation model for complete IP-based SoC system is presented in [13]. It provides an opportunity for designers to select an optimal architecture of a system and also optimize the system power at a higher level of abstraction. Power estimates of dynamic power are compared with existing power models for validity and analysis of speed and accuracy. A comparison of different dynamic power estimation methods is discussed in [14]. A model is tested on three different systems and then a power reduction method is applied to reduce the power estimate at the RTL-level. The clock gating method is used for power reduction at the RTL-level. A multi-mode power estimation methodology for larger designs at the RTL level is presented in [15]. Two major techniques are used in the proposed model. The first one is based on power classification for the refinement of power behavior for larger designs. In the second technique, the weighting function is used for power characterization. An integrated power model based on IP-based macro-modeling and system level for MPSoC is proposed in [16]. An advantage of using these two levels is that we can estimate the power of any multi-core SoC with higher accuracy and time efficiency. In the macro-modeling-based approach, operation, instruction-level, and pipeline-based models are used for validation of results with existing approaches.

A cycle-accurate power model based on machine learning is proposed in [17]. The proposed model is trained on the information based on switching characteristics for accurate power estimation at the RTL level. A LUT-based power macro-modeling at the RTL-level is proposed in [18]. A modified parameter based on ‘switching activity distribution’ is used and also reference table method based on circuit characteristics is used for better accuracy and power estimation. The power model based on the toggle rate for FPGA-based circuits is proposed in [19]. The model is based on the XOR-based decomposition technique and also used the spatial correlation between the patterns. It is validated on different platforms of FPGAs and accuracy can be improved by compressing the computation time of counting the toggle rate and spatial correlations. An IP-based power estimation model for SoC at the system level is proposed in [20]. For fast and accurate power estimation, a model-driven approach is used in annotated-based and standalone power estimators for simulation of various SoC architectures.

An ARM-based power estimation methodology for complex SoC is proposed in [21]. It is dependent upon the statistical power models and regression analysis of individual components of a system. TSV is an essential component for providing vertical interconnects between different cores in 3D IC design. Performance parameters of TSV are its resistance, capacitance, and inductance and they must be modeled to know the effect of these parameters on the performance. A hybrid architecture consisting of CPU, DRAM, and FPGA layers for 3D ICs is proposed in [22]. The CPU layer is placed close to the FPGA layer so that communication between these two layers becomes faster and it can easily access the data caches. It also reduces the system power with higher performance. A macro-modeling approach for homogeneous integration of NoC based mesh architecture is proposed in [23]. The first part of the proposed model estimates the average power consumption of the NoC architecture of each core, while the second part estimates the average power of uniformly distributed TSVs.

### 2.1 Problem formulation

**Fig 1:**
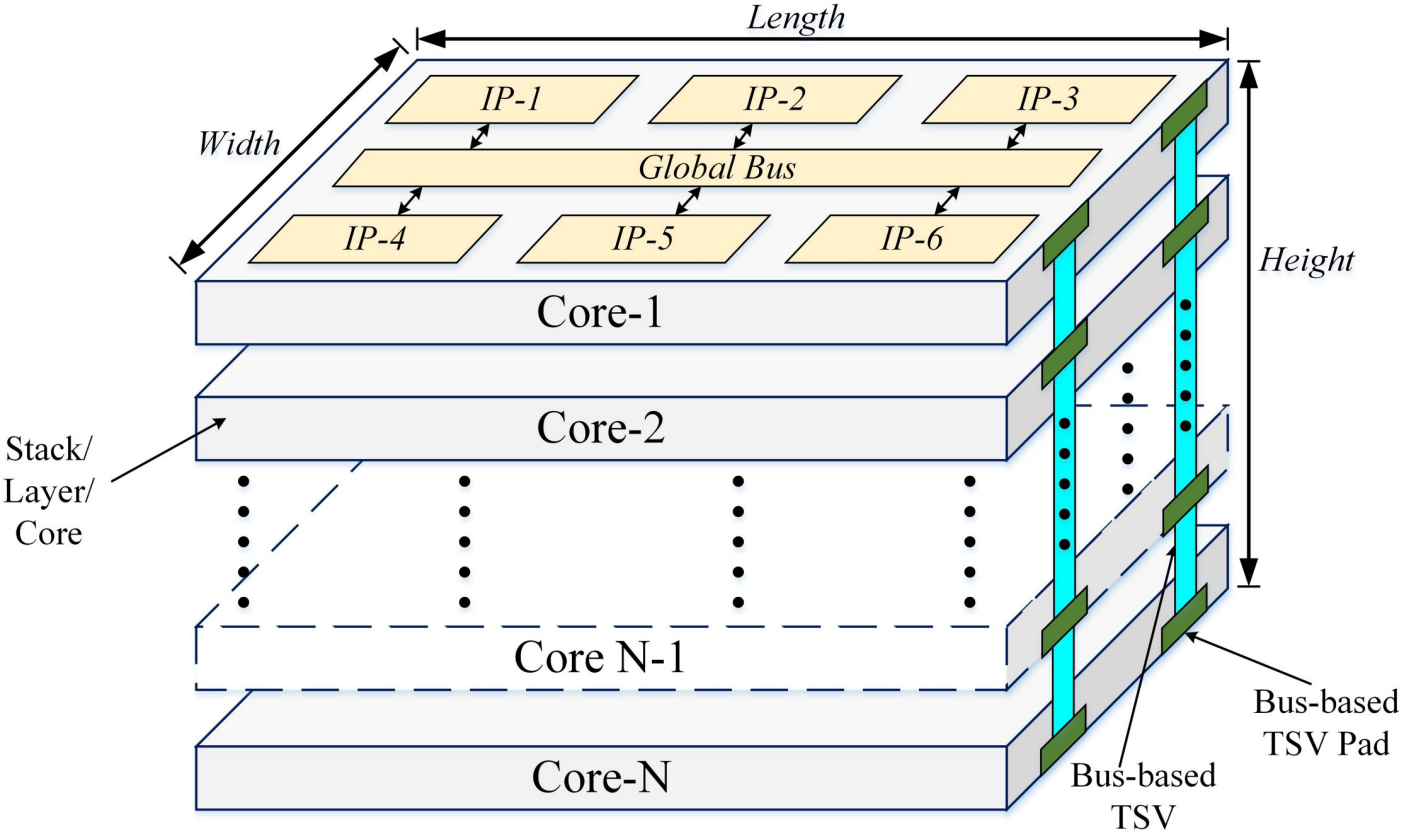
IP-based heterogeneous 3D digital system.

Given a 3D IC with *N* number of heterogeneous layers/dies/cores. Each layer has digital IP blocks with local and global bus interconnections. Multiple cores are connected by bus-based through-silicon-via (B-TSVs). The 3D IC has a dimension of length*width*height. Estimate the average power consumption of this 3D IC as shown in Fig 1.

## 3 Power macromodeling for IP-based 3D digital system

### 3.1 Methodology

One of the most important concerns in the power macro-modeling is the choice of the model’s input parameters. They should capture the primary features that are responsible for the system’s power dissipation and thus it can have good accuracy in its power estimates [24, 25]. We will focus on our problem of statistical power macro-modeling for IP-based 3D digital systems. The entire power estimation method is performed in two major steps. First, the average power is estimated for an IP-based individual cores. Second, the average power for bus-based Through-Silicon-Via (B-TSV) is estimated. Finally, the cores and B-TSVs are integrated together to construct a 3D system. Then the average power for a complete test system is estimated.

Power dissipation is a function of an input signal and its activities, therefore a good power macro-model should consider the accurate activities of the input signals. A simple methodology is to create the LUT that considers the different sets of input patterns and finding the power for these combinations of sets of input patterns. Our improved macro-model consists of a linear function and it estimates the average power dissipation of IP based 3D digital system *P*_3*D*−*digital*−*system*−*avg*_ given in 1:

$$\begin{array}{c} P_{3D-digital-system-avg}=f\left(TD,SP,SV\right) \end{array}$$ (1)

Where *P*_3*D*−*digital*−*system*−*avg*_ is the average power consumption for 3D digital system. The function in the above relation maps the statistical input properties to the power dissipation of the 3D digital system. As power dissipation is a function of input signal characteristics, power estimation is a fast and efficient method. The test system is simulated by applying input vector streams with *TD*, *SP* and *SV*. Given a 3D digital test system having *r* number of inputs of length *s* for binary stream *q* is given in 2:

$$\begin{array}{c} q=[(q_{11},q_{12},\ldots .q_{1r}),(q_{21},q_{22},\ldots .q_{2r}),\ldots .(q_{s1},q_{s2},\ldots .q_{sr})] \end{array}$$ (2)

The input statistical properties are defined for input binary stream defined in above relation are given by 3, 4 and 5 respectively:

$$\begin{array}{c} TD=\frac{\sum_{j=1}^{r}\sum_{i=1}^{s-1}q_{ij}⊕q_{i+1,j}}{r*(s-1)} \end{array}$$ (3)

$$\begin{array}{c} SP=\frac{\sum_{i=1}^{r}\sum_{j=1}^{s}\left(q_{ij}\right)}{\left(r*s\right)} \end{array}$$ (4)

$$\begin{array}{c} SV=\frac{\sum_{i=1}^{r}\sum_{j=1}^{s}{\left(q_{ij}-\;SP\right)}^{2}}{(r*s)-1} \end{array}$$ (5)

Where *TD* is the transition density, *SP* is the signal probability and *SV* is the signal variance of input patterns. *TD*, *SP* and *SV* cover all the aspects of the proposed model and its accuracy will also be proved by experimental results. We propose the addition of a new statistical parameter *SV* and its relation with *SP* and correlation is represented in the next section. These parameters also define the correlation factor between the input patterns. They affect the power dissipation of any IP-based digital system. The proposed model also reduces the computational complexity of the previous model. Therefore, accurate modeling of parameters is required for better accuracy and the exact behavior of the digital system.

### 3.2 Relation between signal variance and signal probability

The signal variance expression is given in Eq 5. It is dependent upon the value of *SP*. Therefore the relation between *SV* and *SP* is given in Eq 6.

$$\begin{array}{c} SV=4*SP(1-SP) \end{array}$$ (6)

**Fig 2:**
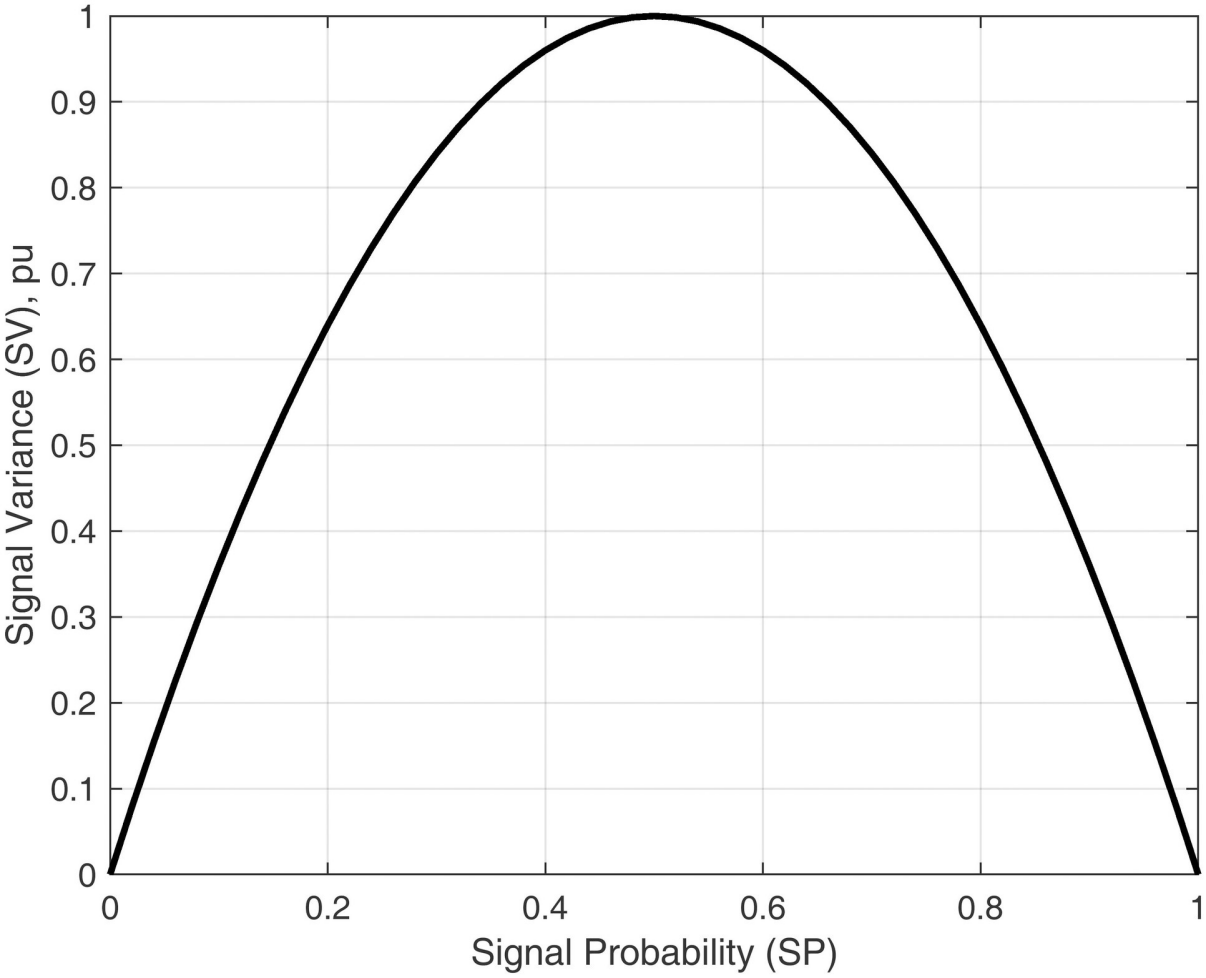
Relationship between signal variance and signal probability.

The graphical representation of the above relation is shown in Fig 2.

The signal variance and signal probability vary in the range of [0 1]. The SV rises parabolically for the values of SP from 0 to 0.5 and then also decreases parabolically from 0.5 to 1. At *SP* = 0.5, the value of *SV* is maximum. At the extreme values of *SP* ([0 0.2] and [0.8 1]), the *SV* is minimum.

### 3.3 Relation between signal variance and signal correlation

Our proposed macromodel have 3 parameters *TD*, *SP* and *SV*, instead of correlation parameters *S*_*c*_ and *T*_*c*_ [23, 26, 27]. *S*_*c*_ and *T*_*c*_ are spatial and temporal correlations. Our model is more efficient because it has fewer input parameters as compared to the previous model. This will be proved in the discussion of experimental results. Our new statistical input parameter *SV* has also significant relation with Signal Correlation. The relation between them is given as:

$$\begin{array}{c} SV=-0.5*SC+0.5 \end{array}$$ (7)

*SV* and *SC* varies in the range of [0 1]. It has a negative slope as evident from Eq 7. From this relation, if we have a value of *SC*, we can find the value of *SV*. Therefore by running the full span of input properties, we can find the power estimates at the specific values of input sets. Also if we include correlation parameters, redundant simulations will be added, because we already incorporated the effect of correlation in the form of signal variance in the proposed model. First of all, optimized input patterns are generated by using the optimization algorithm explained in the next subsection.

### 3.4 Input pattern generation using Biogeography-Based Optimization (BBO)

BBO is established by Dan Simon [28] and it is a biogeography-based optimization algorithm. Probabilistic models of biogeography are used in the formulation of this optimization algorithm. It is also used in the problem of renewable energy integration for the least cost of operation [29, 30]. Biogeography is defined as the behavior of different species to migrate between different habitats. Generally, it includes speciation, migration, and extinction of species. Migration of different species has two major types: emigration (Non-suitable habitats) and immigration (suitable habitats). It has four types of population-based habitats. Individual habitat is defined as a search agent and it is defined by the parameter of suitability index variable (SIV). Decision values for K number of habitats having M dimension for SIV are given by:

$$\begin{array}{c} \begin{array}{c} H_{k}=[\begin{array}{ccccc} N_{1}^{k} & N_{2}^{k} & N_{3}^{k} & \cdots & N_{M}^{k} \end{array}] \end{array} \end{array}$$ (8)

$$\begin{array}{c} \begin{array}{c} P_{B}={\left[\begin{array}{ccccc} H_{1} & H_{2} & H_{3} & \cdots & H_{M} \end{array}\right]}^{T} \end{array} \end{array}$$ (9)

$$\begin{array}{c} P_{B}=[\begin{array}{cccc} N_{1}^{1} & N_{2}^{1} & \cdots & N_{M}^{1}\\ N_{1}^{2} & N_{2}^{2} & \cdots & N_{M}^{2}\\ \vdots & \vdots & N_{m}^{k} & \vdots \\ N_{1}^{K} & N_{2}^{K} & \cdots & N_{M}^{K} \end{array}], \end{array}$$ (10)

where *m* = {1, 2, 3…*M*}, *k* = {1, 2, 3…*K*},*P*_*B*_ is the population matrix of search agents and $N_{m}^{k}$ is the *m*^*th*^ value of decision variable for *k*^*th*^ agent.

The performance of each habitat is evaluated by its fitness value and also called habitat-suitability-index (HSI). The computation of HSI values of habitats is given by following relation:

$$\begin{array}{c} \begin{array}{cc} C_{B}(k) & =f_{obj}(H_{k}),\;\forall k,\\ & =f_{obj}(N_{1}^{k},\;N_{2}^{k},\;N_{3}^{k},\;\cdots N_{M}^{k}), \end{array} \end{array}$$ (11)

where *C*_*B*_(*k*) is the cost function of *k*^*th*^ search agent. On the basis of HSI, the population is sorted and the habitat having the least value is declared as the best habitat. The mutation is performed on different habitats for the specified number of generation of new habitats. Migration operator gives useful information of habitats by using immigration and emigration aspects. The calculation of these aspects for *k*^*th*^ habitat as given by:

$$\begin{array}{c} \lambda_{k}=I\times (1-\frac{Q_{k}}{Q}), \end{array}$$ (12)

$$\begin{array}{c} \mu_{k}=E\times (\frac{Q_{k}}{Q}), \end{array}$$ (13)

where λ_*k*_ is the immigration rate of *k*^*th*^ search agent, I is the maximum immigration rate, *μ*_*k*_ is the emigartion rate for *k*^*th*^ agent, E is the maximum emigration rate, Q is the maximum number of species and *Q*_*k*_ is the number of species for *k*^*th*^ habitat. Different models are used in literature for the calculation of Immigration and emigration rates. For simplicity, we consider the linear migration model (E = I) here.

The initial population contains a greater amount of habitats having higher values of HSI. Therefore lower-valued HSI habitats tend to adopt the features of high-immigration and lower-emigration rates habitats. The high-valued HSI habitats ensure that low-valued HSI habitats adopt good features in them or not.

**Fig 3:**
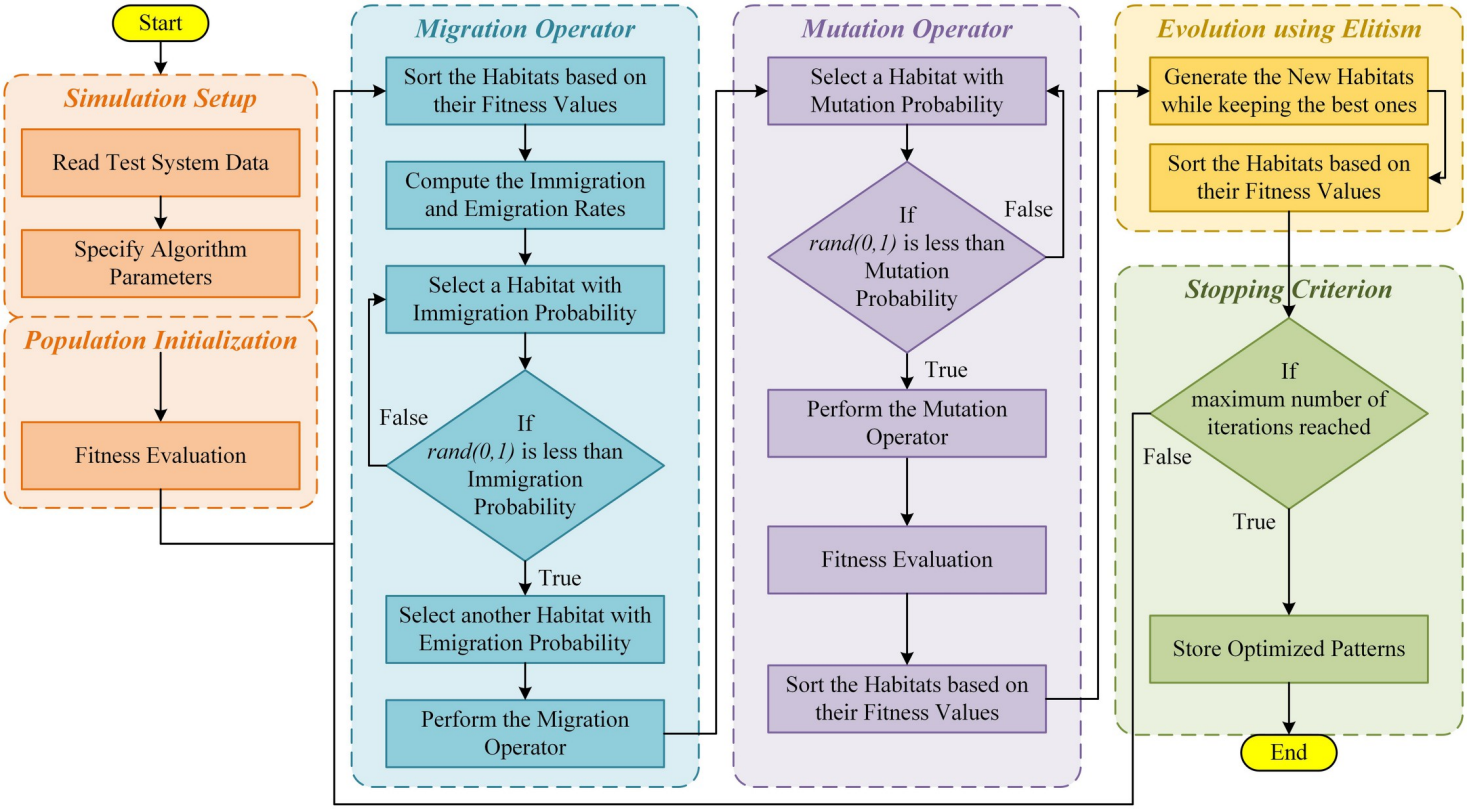
Flow chart of BBO.

**Fig 4:**
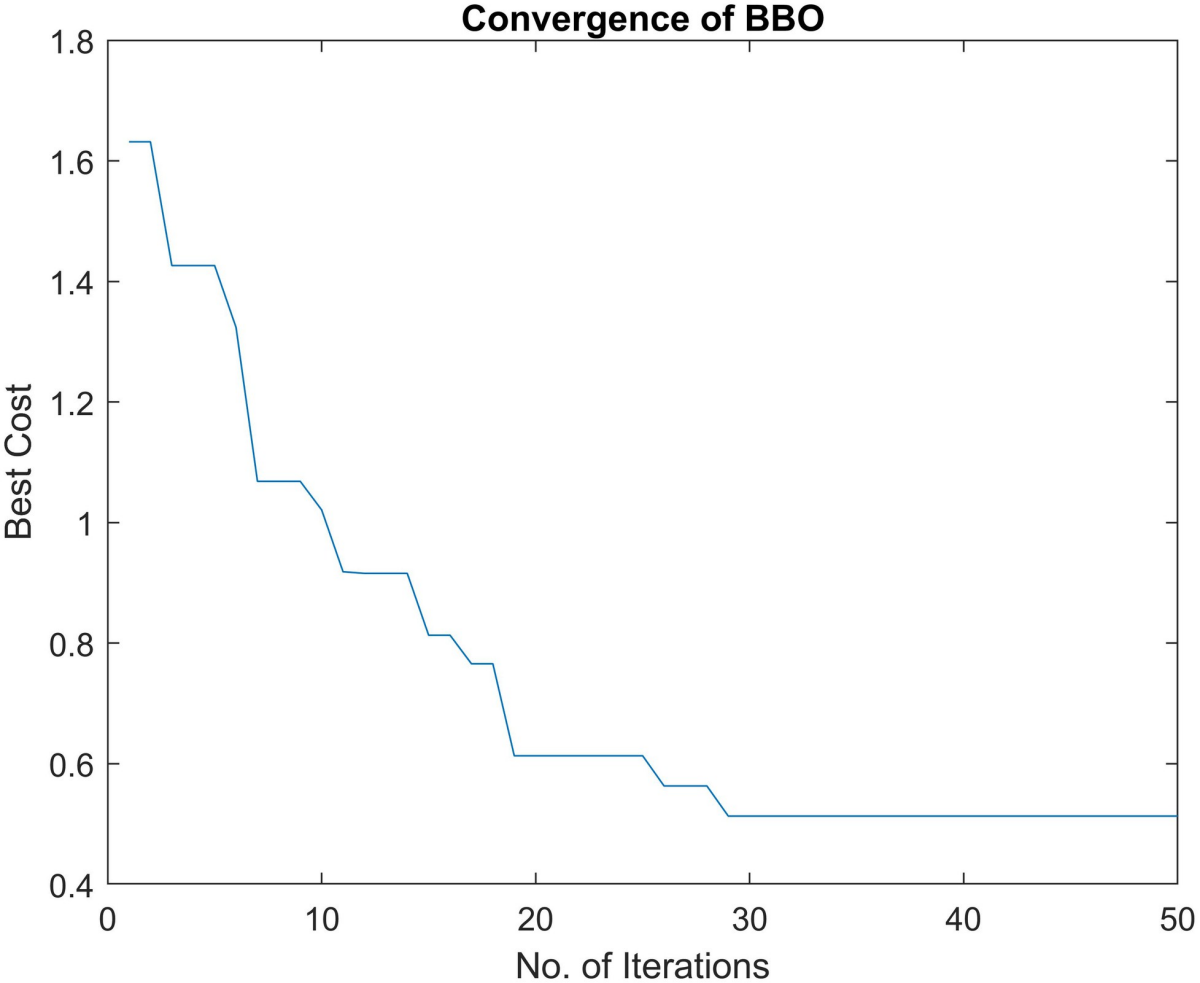
Convergence of BBO.

The above-mentioned exploitation behavior of BBO is well established and strong so that it searches the entire space effectively and also used elitism behavior. Migration of individual habitat is given in the following relation:

$$\begin{array}{c} H_{k}\leftarrow H_{q}, \end{array}$$ (14)

where *H*_*k*_ is the immigrating habitat selected based on immigration rate and similarly *H*_*q*_ is the emigrating habitat based on good emigration rate. Whereas the selection of *q*^*th*^ search agent is done by Roulette-wheel criteria. These search agents are used for the generation of new search agents and given by:

$$\begin{array}{c} {N_{new}}_{m}^{k}=N_{m}^{k}+\alpha_{s}\times \left(N_{m}^{q}-N_{m}^{k}\right) \end{array}$$ (15)

where *N*_*new*_ is the newly generated search agents, *α*_*s*_ is the step size. In most of the optimization algorithms, during the iterations, the rate of solution stuck in local-minimum is relatively high. To solve this issue, mutation operators help BBO to avoid trapping and also implementing the space exploration. Probabilistically, a Mutation operator works on the basis of mutation rates of each habitat. Mutation of a new generation of these search agents is performed as given by:

$$\begin{array}{c} {N_{new}}_{m}^{k}={N_{new}}_{m}^{k}+\sigma_{s}\times \text{rand} \end{array}$$ (16)

where *σ*_*s*_ = 0.02× (*N*_*max*_ − *N*_*min*_) and *rand* is random number generated. *σ*_*s*_ is the mutation step size, *N*_*min*_ and *N*_*max*_ is the minimum and maximum of search agents. Etilist parameter is used for the updation of population matrix and it is given in relation:

$$\begin{array}{c} {P_{B}}_{new}=[\begin{array}{cccc} N_{1}^{1} & N_{2}^{1} & \cdots & N_{M}^{1}\\ \vdots & \vdots & \ddots & \vdots \\ N_{1}^{nk} & N_{2}^{nk} & \cdots & N_{M}^{nk}\\ {N_{new}}_{1}^{1} & {N_{new}}_{2}^{1} & \cdots & {N_{new}}_{M}^{1}\\ \vdots & \vdots & \ddots & \vdots \\ {N_{new}}_{1}^{nn} & {N_{new}}_{2}^{nn} & \cdots & {N_{new}}_{M}^{nn} \end{array}], \end{array}$$ (17)

whereas *K* = *nk* + *nn*. *P*_*B*__*new*_ is the newly generated population matrix, nk and nn are the number of kept and new search agents. The process of evolution of habitats is terminated on reaching the maximum number of iterations programmed. For independent test runs of an optimization problem, the simulation parameters of BBO are tuned to the values that are specified in Table 1. Similarly, the flowchart of BBO is shown in Fig 3. The convergence of BBO for a typical test example is shown in Fig 4. The pseudo-code of BBO is given as below:

**Table 1:** Simulation parameters of BBO.

Sr. No.	Parameter	Value
1	Keep Rate	20%
2	*α*_*s*_	90%
3	*σ*_*s*_	2%
4	Mutation Probability	10%
5	Population Size	30
6	Number of Iterations	50

### 3.5 Monte-Carlo simulation

With the increase of cores in a 3D digital system, the number of inputs required for each core is also increasing exponentially. For testing of each input vector, the simulation time and complexity of the circuit is also increased. There are mainly two approaches discussed in the literature for reducing the time consumption in power estimation. The first one is to generate the shorter input vectors and the other one samples the portion of samples from the originally generated input vectors. For the regeneration of input patterns having a shorter length from the original ones that have the same average values of power consumption and it also preserves the input characteristics of the macro-model [23]. The Monte Carlo simulation approach is used for power estimation of circuits is proposed in [31, 32]. This method uses the original samples of input vectors and a simulation method is used for deriving the average value of samples which is used for finding the average value of the power estimation. From the Central Limit Theorem, the sample value of power estimates assumes the normal distribution with length l approaches infinity. If the random variable *x* is not approaching the normal distribution, the basic assumption is not satisfied, thus it may have a large error percentage.

**Fig 5:**
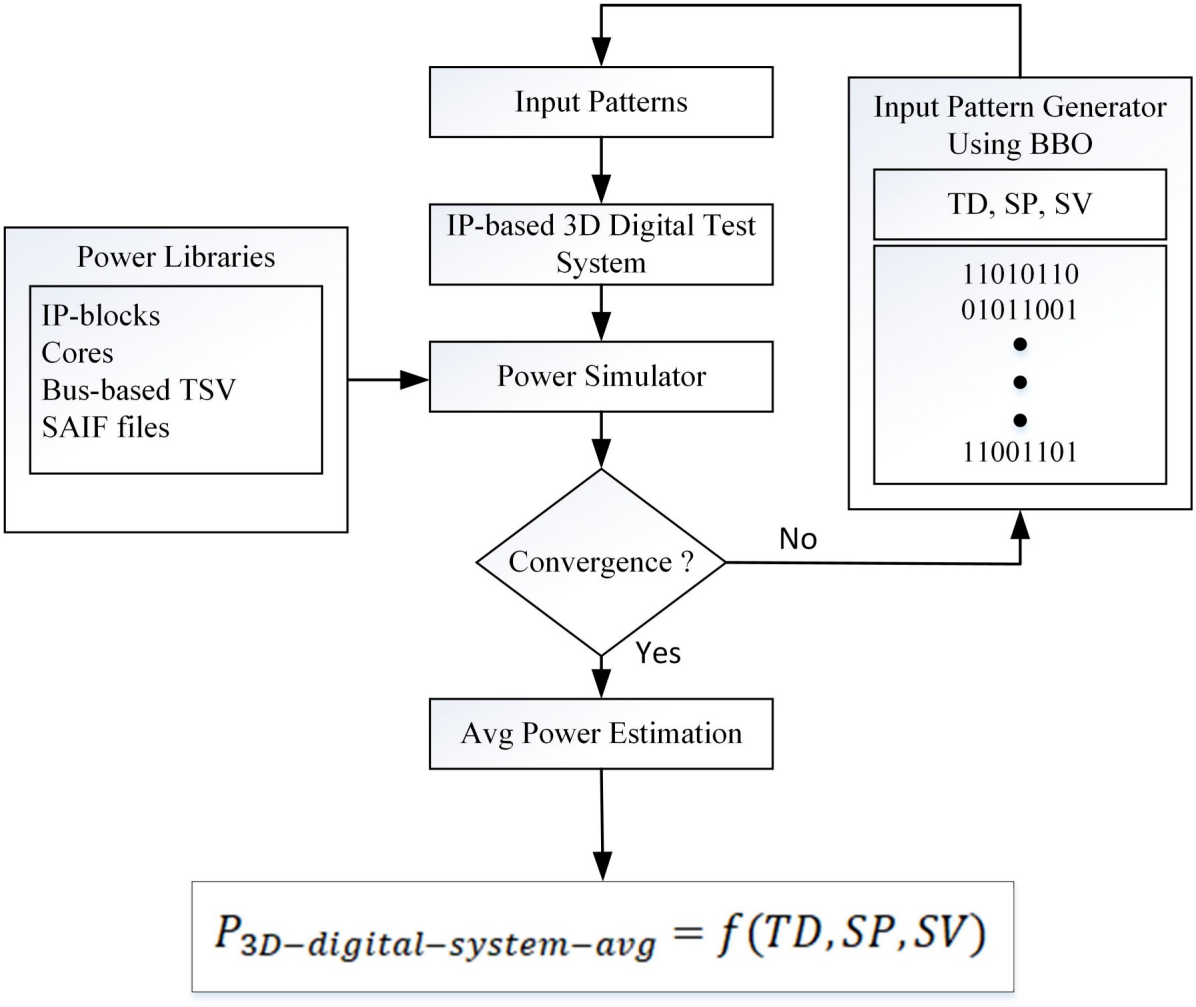
Proposed methodology.

In this research, we use the IP-based statistical sampling technique that is based on the Monte Carlo Simulation approach. Each IP module is described at the RTL-level and the power estimation technique is also applied at the same level of abstraction. The proposed approach aims to estimate the average power consumption of each IP module under the user-specified input stream. It is estimated under the user-defined confidence interval and level of error percentage. It is very important to find the accurate input patterns and also the number of simulations required for the convergence of power samples. The smaller sample granularity increases the overall efficiency of this approach. Two points should be considered for selection and the numbers of simulations for convergence are: Reduce sample variance Small sample granularity for achieving near-normal distribution of samples Fig 5 explains the flow of the proposed methodology. The simulation is performed in every iteration. The circuit under test is simulated by applying the input vector stream of predefined statistical characteristics. The power results are monitored and sample mean and variance are calculated. The process terminates if it meets the predefined stopping criteria for simulation.

**Algorithm 1**: Pseudo Code of BBO

**Input**: BBO Parameters: *α*_*s*_, *σ*_*s*_, mutation probability, keep rate (elitist parameter), maximum iterations and population size

**Output**: Store optimized input patterns

1 Define immigration rates

2 Define emigration rates

3 Initialize the random population of *K* habitats

4 Compute fitness (HSI) of each habitat

5 Sort the habitats based on their fitness values. The first habitat will be the best solution

6 **while** *iter* < *maximum iterations* **do**

   /* BBO Operators       */

7   **for** *k* = 1: *K* **do**

8    Select *H*_*k*_ with probability λ_*k*_

9    **for** *m* = 1: *M* **do**

    /* Habitat Migration       */

10     **if** *rand*_*real*(0 ∼ 1) < λ_*k*_ **then**

11      Select *H*_*q*_ with probability *μ*_*k*_

12      Migration operator is performed to generate a new population of habitat $H_{k}^{new}$

13     **end**

     /* Habitat Mutation       */

14     **if** *rand*_*real*(0 ∼ 1) < mutation probability **then**

15      Mutation operator is performed on $H_{k}^{new}$

16     **end**

17    **end**

18    Compute fitness (HSI) of $H_{k}^{new}$

19   **end**

20   Sort the new population of habitats based on their fitness values

21   Population of habitats *P*_*new*_ is formulated for next iteration

22   Sort the new population of habitats based on their fitness values

23   Find the best solution among the habitats

24 **end**

The input pattern generator is based on BBO, it generates the input waveforms according to these predefined statistical properties as mentioned in section 3. These waveforms are applied to the power simulator to derive the sample values of power estimation. At a given time *T*, the power simulator gives the value of the IP module under test is called a sample value. If this process is repeated *N* times, the average of these values for *N* trials is called sample mean. For larger values of *N*, the sample mean approaches towards the true mean, and that criteria are known as stopping criteria.

Where *μ*_*m*_ is the average power consumption and *N* is the number of simulations. Similarly Power(*V*^*i*^) is the power consumption when the input patterns make transitions from *i*^*th*^ to (*i* + 1)^*th*^ input pattern. Let *P* be the random variable that contains all the power values of *Power*(*V*^*i*^). If we take the average of *N* samples;is called sample *m*, and its mean is desirable that it approaches the true average power *μ*_*m*_.

According to the Central Limit Theorem, the random variable *m* has a distribution closer to normal distribution for a larger value of *N*. Therefore to find the correct value of sample mean, the MCS helps us to estimate the correct value of average power without simulating all the input patterns.

$$\begin{array}{c} P(\frac{\mu_{m}-\bar{m}}{\bar{m}})\leq \varepsilon =1-2\alpha ,0\leq \alpha \leq 0.5 \end{array}$$ (18)

Where *ε* is the error and *α* is the confidence level. This equation explains that the user has a confidence interval of (1 − 2*α*) that the error between the real and sample mean is less than *ε*. If an error is greater than a user-defined error, then more samples are required from *N* to repeat the evaluation and it repeats until the error is less than the user-defined error interval.

### 3.6 Power modeling for IP-based individual core

The function described in Eq 1 is a LUT-based method that is used in the simulation and characterization phase of input patterns. To find the accurate function and their dependency, we must generate several input pattern streams for each core having IP blocks and buses, defined in Eq 19:

$$\begin{array}{c} P_{core-avg}=f_{1\to N}\left(TD,SP,SV\right) \end{array}$$ (19)

In the above relation, *P*_*core*−*avg*_ is the average power dissipation for a single core. *N* is the number of cores in a IP-based 3D digital system.

We aim to find the average total power dissipation of IP blocks and buses by applying user-specified input patterns at a single core level. Power estimation is done by using the Monte Carlo simulation approach so that a high-level power estimate satisfies the user-specified confidence interval. We sum up the estimates of all IP blocks and buses for finding the total power consumption of a single core.

### 3.7 Power macromodeling for Bus-based Through-silicon-via (B-TSV)

**Fig 6:**
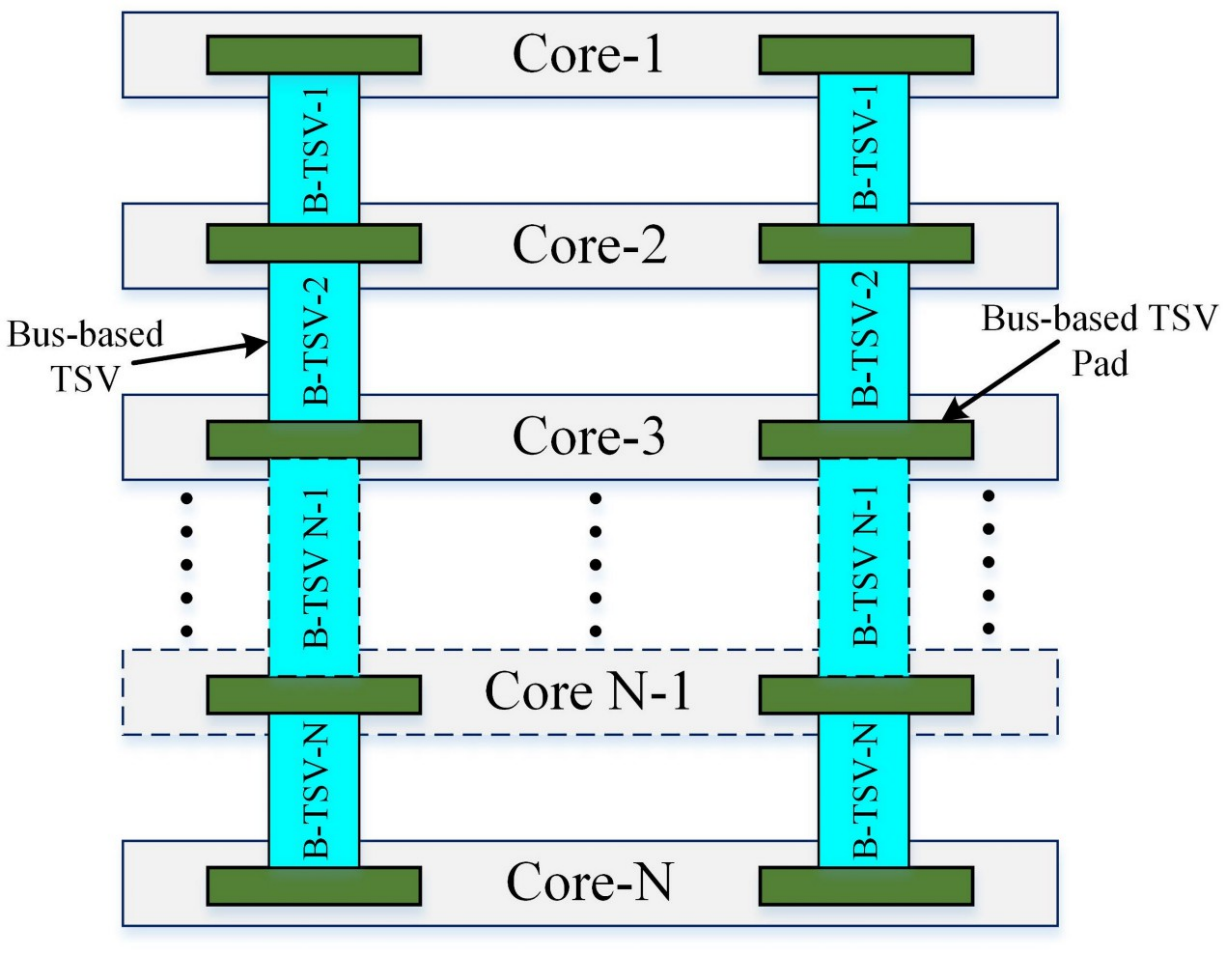
Bus-based TSV.

Various power modeling techniques for interconnects/buses have been discussed in [33, 34]. We have used the same statistical power macro-model described in 1 for the calculation of average power consumption for B-TSVs. The concept of B-TSV is explained in Fig 6. It can have *N* number of B-TSVs which are used for communication between cores in a 3D IC. The relation for power consumption of B-TSVs is shown in 20:

$$\begin{array}{c} P_{B-TSV-avg}=f_{1\to N}\left(TD,SP,SV\right) \end{array}$$ (20)

In the above relation, *P*_*B*−*TSV*−*avg*_ is the average power dissipation for B-TSVs. *N* is the number of B-TSVs in a IP-based 3D digital system.

### 3.8 Power modeling for IP-based multi-Core 3D digital system

The function described in Eq 19 is a power macro-model for a single core with buses. We will use the same function described in 1 to find the power estimation for a multi-Core 3D digital system. A power model for IP-based multi-Core 3D digital system with B-TSVs is defined in Eq 21:

$$\begin{array}{c} P_{3D-Digital-system}=\sum_{i=1}^{N}P_{i_{core-avg}}+\sum_{i=1}^{K}P_{i_{B-TSV-avg}} \end{array}$$ (21)

In the above relation, *N* is the number of single cores and *K* is the number of B-TSVs in IP-based multi-core 3D digital system.

## 4 Experimental results

In this section, we will discuss our experimental results of the proposed macro-modeling approach. The accuracy of the proposed model is evaluated on IP-based 3D digital system. The different experimental setup has been designed to study the accuracy and efficiency of our proposed approach for average power estimation of a complete 3D IC. We will present the heterogeneous integration of different cores which are connected by the B-TSVs. The following criteria’s are used for the validation of our experimental results:

1. Validation of Regression-based model for IP-based heterogeneous 3D IC
2. Accuracy test with Vivado HLS power estimation results
3. Evaluation of proposed model with existing models

A detailed discussion on experimental results and error analysis is given in the following subsections.

### 4.1 Power macro-model validation

For the demonstration of the accuracy of our proposed model, we performed several experiments on different IP-based digital test systems. We have implemented these systems at the RTL-level. In the power estimation phase, several input patterns are generated with statistical characteristics of *TD*, *SP*, *SV* for individual IPs, buses, B-TSVs and heterogeneous integration of cores by using BBO. By using the LUT approach, functional simulations at the RTL-level are performed with power simulators to find the average power dissipation of each IP-based core, B-TSV and 3D system. A Monte-Carlo zero-delay simulation is performed with several input pattern characteristics for accurate power estimation. Convergence analysis is performed to find the accurate power estimates so that it satisfies the defined level of a confidence interval. Linear regression and statistical analysis are performed to find the quality of the model’s fit and accuracy. Power macro-modeling results are compared with Vivado HLS. In the end, the average power errors and their fitting are computed.

For the verification of randomly generated sequences *P*_*Simulated*_ and *P*_*Estimated*_ are compared and found correlation factors with a range from 95–99% for each IP block, bus, heterogeneous integration of cores and B-TSVs. Several input sequences of 4, 8, 16, 32 bits are generated. A low-power IP-based 3D digital system is implemented and verified @100 MHz operating frequency. The simulations for the test system are performed on Desktop PC: Make HP, Intel Core i7 @ 3.4 GHz processor, 8 GB RAM and 64-bit operating system (Windows 10). All the sequences are uniformly generated with a 95% confidence interval (*α* = 0.05) with 5% error tolerance over the entire space of [0–1].

### 4.2 Model accuracy analysis

The proposed pattern generator uses BBO for the generation of sets of sequences over the full span of [0 1]. This feature of the pattern generator enables us to find the accurate relationship between the average power and input signal statistics. In our experiments, we designed different cores to make a 3D digital test system. For each of the IP blocks, we generated distinct 2250 sequences with *TD*, *SP* and *SV* evenly distributed in 3-D space. The granularity of 0.1 is used for the entire test system. In practice, more sequences are required for the larger circuits for the steady-state average power. For an IP block, large input sequences will produce similar steady-state power also exhibits the same total power. That average power corresponds to input sequences that have similar steady-state power have a behavior of a random variable. Then simulation is performed for each IP block to obtain power dissipation at RTL level with the generated inputs. It is observed that for a fine granularity for *TD* and a large granularity for *SPandSV* in the characterization step, we obtain the more accurate results and it also reduces the characterization time of the model.

### 4.3 IP-based digital test system-I (Core-1)

The proposed macro-model accurately estimates the average power of a 3D digital system. To further validate our model, we construct the IP-based test system by summing up the powers for individual IP blocks and buses. The test system-I is discussed in our previous work for IP-based single-core [25]. The test system is a 32-bit MIPS architecture having a different(six) number of IP blocks that are connected by different buses. In this section, we will demonstrate only the accuracy of the power estimates of this test system.

**Fig 7:**
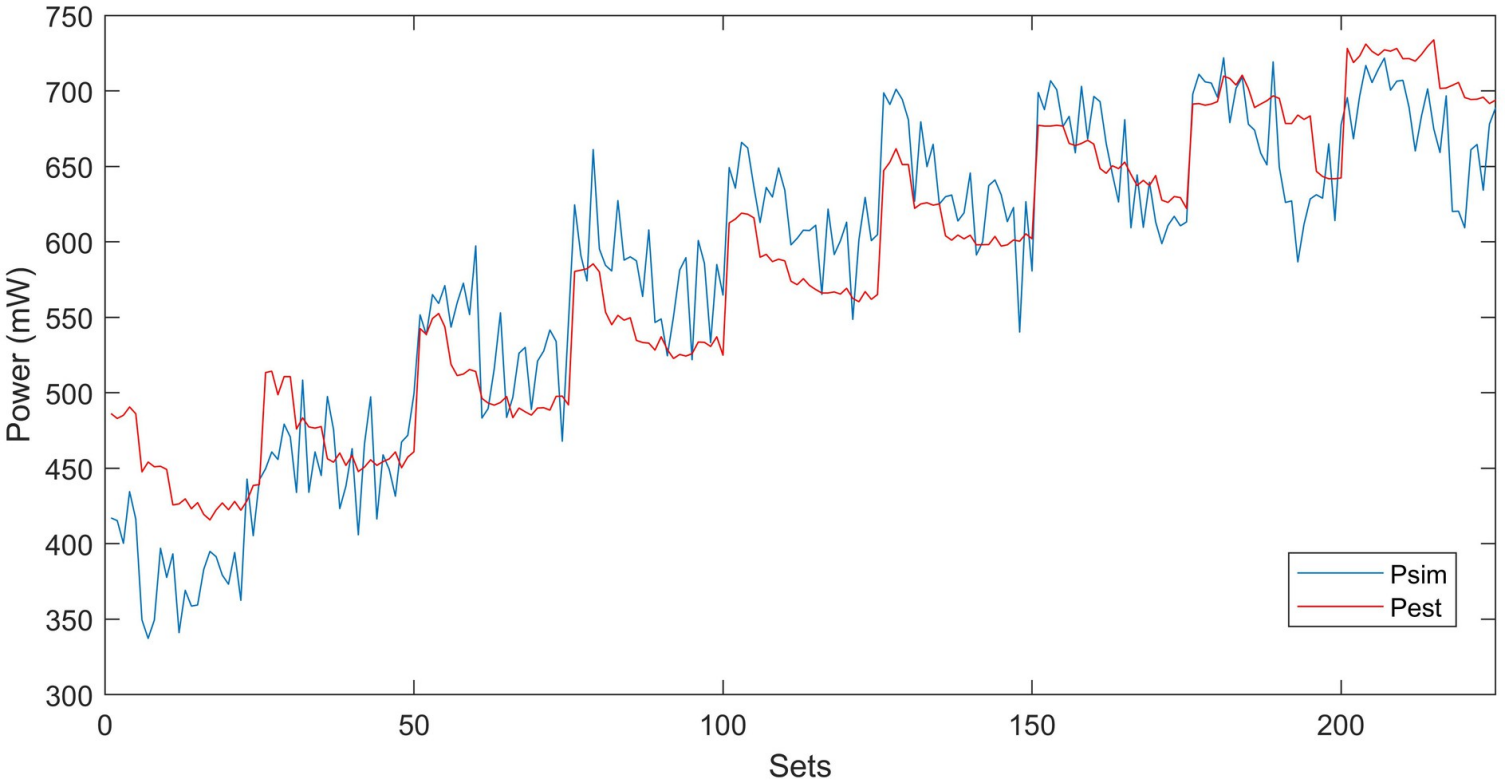
Correlation between simulated and estimated powers for test-system I.

By using the proposed model, computational complexity is reduced, because we reduce the input variables. Therefore, the extensive amount of simulations are also reduced to minimize the computational time with better accuracy of results. Linear regression analysis is performed to find the quality of the model’s fit and accuracy. The accuracy of the proposed macro-model is tested by running RTL simulations. Power macro-modeling results are compared with Vivado HLS. In the end, the average power errors and their fitting are computed. Correlation between estimated and simulated powers is shown in Fig 7. Different IP blocks are connected with local and global buses to construct an IP-based SoC digital system. The proposed macro-model makes the power estimation an easy task for designers by simply adding the power estimates of IPs and buses. It also helps designers to reuse and explore different complex IP blocks in real-time in a plug-and-play fashion. By using the power macro-modeling approach, a power estimate for any IP-based SoC system can be found easily by the simple addition of power estimates of individual IP blocks. The estimated power *P*_*SoC*−*system*_ in Eq (1) is compared with simulated power *P*_*Simulated*_ (Vivado HLS Simulator) for the evaluation in accuracy of power estimation.

Due to randomly generated input patterns, power estimates of the macro-model have certain uncertainties. To give valid conclusions, error analysis should be performed based on experimental results. For individual IP-based single-core, 225 distinct sequences are generated with *TD*, *SP*, *SV* over the entire space of [0–1]. An extensive number of simulations for each block helps us to reveal the accurate relationship of power estimates with input characteristics. The full span of input characteristics is tested to model the real-time scenarios of the system. It is observed that our macro model is more accurate between the span of [0.2 0.8] and less accurate for the span of [0 0.2] and [0.8 1]. For the verification of randomly generated sequences *P*_*Simulated*_ and *P*_*Estimated*_ are compared and found correlation factors with a range from 95–99% for each IP block/bus.

For the calculation of average absolute error of each IP block/core/B-TSV/3D IC, the relation is given in Eq 22:

$$\begin{array}{c} E_{avg}=\frac{1}{N}\sum \{\frac{|P_{simulated}-P_{estimated}|}{P_{simulated}}\} \end{array}$$ (22)

Where *E*_*avg*_ is the average absolute error, *N* is the number of simulations.

All the sequences are uniformly generated with a 95% confidence interval over the entire space of [0–1]. In Table 2, the 2nd column represents the name of core, the 3rd to 5th column represents the maximum, average and minimum power, whereas errors are demonstrated in columns 6th to 8th. Results presented below demonstrate that our power macro-model gives designers an early and accurate estimate of power for an IP-based single-core. We have used BBO for the generation of improved and optimized input patterns with pre-defined statistical characteristics having full span coverage [0 1] to improve the accuracy of the previous macro-model.

**Table 2:** Power estimates and accuracy for test-system I.

Sr. No	Name of Core	P_max_ (mW)	P_avg_ (mW)	P_min_ (mW)	E_max_ (%)	E_avg_ (%)	E_min_ (%)
1	Core-1	721.9	595.74	373.2	17.33	5.14	0.018

For test system-I, an average error of the individual core-1 is measured 5.14% as shown in Table 2.

**Fig 8:**
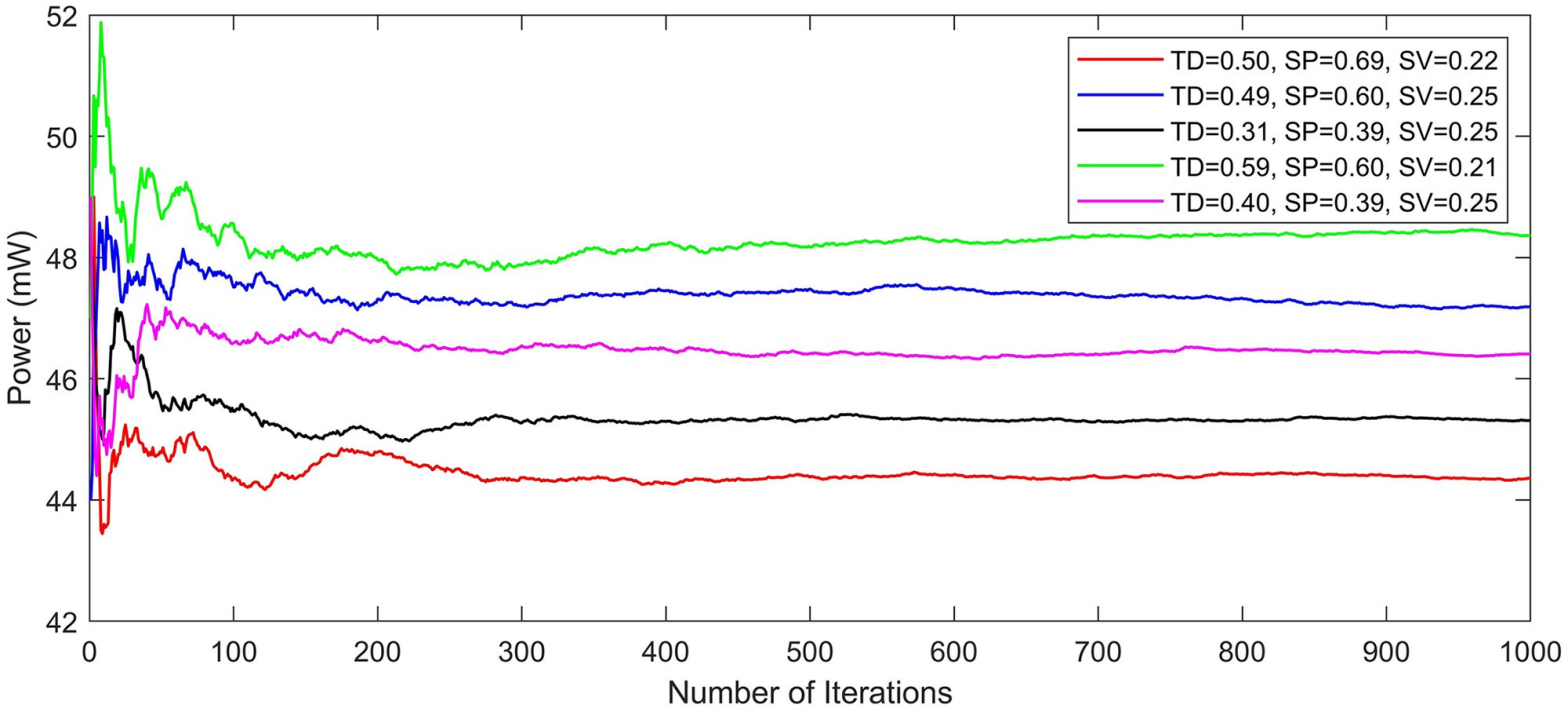
Convergence analysis of IP-2 (core-1) using Monte-Carlo simulation.

This error can be further improved by the model for propagation delay, glitches, and jitters, etc. For simplicity, we assume our macro-model with a zero-delay approach. For IP block-2 of this test-system, by using the Monte Carlo simulation approach, as shown in Fig 8, the first 400 iterations are called the warm-up region. Then from 500 to 700, this region is called the steady-state region and we take the average of steady-state power values. Results presented below demonstrate that our power macro-model gives designers an early and accurate estimate of power for an IP-based digital system.

### 4.4 Digital test system-II (Core-2)

In this section, we will demonstrate the power estimates and their accuracy for IP-based Test system-II. We will also present the experimental results of our proposed macro-model for power estimation as given in Table 3.

**Table 3:** Power estimates and accuracy for test-system II.

Sr. No	Name of IP	P_max_ (mW)	P_avg_ (mW)	P_min_ (mW)	E_max_ (%)	E_avg_ (%)	E_min_ (%)
1	Core-2	86.6	78.93	71.6	9.13	3.56	0.004

### 4.5 Digital test system-III (Core-4)

For the evaluation of our proposed model, we take another digital test system-III. The size of this test system vary from 8 to 32 bits. It has four IP blocks that are connected by different buses. The experimental results of each of the IP block of this core is explained in this section. All the sequences are uniformly generated with a 95% confidence interval over the entire space of [0–1]. In Table 4, the 2nd column represents the type of IP Block, 3rd to 5th column represents the maximum, average, and minimum powers. Maximum, average, and minimum errors are demonstrated in the 6th to 8th columns. Results presented below demonstrate that our power macro-model gives designers an early and accurate estimate of power for an IP-based digital system-III.

**Table 4:** Power estimates and accuracy for test-system III.

Sr. No	IP-block	P_max_ (mW)	P_avg_ (mW)	P_min_ (mW)	E_max_ (%)	E_avg_ (%)	E_min_ (%)
1	IP-1	6	5.61	5	7.08	2.27	0.069
2	IP-2	104	67.05	28	13.02	7.23	0.013
3	IP-3	6	5.62	5	11.77	3.05	0.023
4	IP-4	165.1	108.95	55.1	17.23	10.06	0.073
Average					12.27	5.65	0.04

### 4.6 Bus-based Through-silicon-via (B-TSVs)

In this section, we will demonstrate the power estimates and their accuracy for different sizes of B-TSVs. As discussed earlier, the B-TSV plays an important role in the 3D integration of Cores. After discussing the experimental results of B-TSV, we will combine the heterogeneous cores with B-TSVs to make a 3D IC. We will also present the experimental results for heterogeneous integration for the validation of our proposed macro-model for power estimation of 3D ICs.

We used the same function described in Eq 1 for the generation of optimized input patterns for the simulation of B-TSV at the RTL-level. Table 5 presents the power estimates and their accuracy for the different sizes of B-TSV. It is evident from the experimental results that our model also efficient results in terms of accuracy for B-TSV just like the digital test system.

**Table 5:** Power estimates and accuracy for B-TSVs.

Sr. No	Name of IP	P_max_ (mW)	P_avg_ (mW)	P_min_ (mW)	E_max_ (%)	E_avg_ (%)	E_min_ (%)
1	B-TSV (16-bits)	228	155.93	83	2.17	0.64	0.0078
2	B-TSV (32-bits)	427	316.10	207	5.07	1.13	0.0042

### 4.7 IP-based 3D digital test system

In this section, we will demonstrate the power estimates and their accuracy for heterogeneous integration of multiple cores with B-TSVs. After discussing the experimental results of B-TSVs in the previous section, we will combine the IP-based cores with B-TSVs to make a complete 3D IC. We will also present the experimental results for this type of integration for the validation of our proposed macro-model for power estimation of 3D ICs.

We used the same function described in Eq 1 for the generation of optimized input patterns for the simulation of heterogeneous 3D ICs at the RTL-level. Table 6 presents the power estimates and their accuracy for the complete 3D IC. It is evident from the experimental results that our model also efficient results in terms of accuracy for heterogeneous 3D IC just like the IP-based digital systems.

**Table 6:** Power estimates and accuracy for IP-based heterogeneous 3D IC.

Sr. No	3D IC	P_max_ (mW)	P_avg_ (mW)	P_min_ (mW)	E_max_ (%)	E_avg_ (%)	E_min_ (%)
1	Heterogeneous (4 Cores)	2521	1978	1336	17.40	8.65	0.023

### 4.8 Multiple regression analysis of heterogeneous 3D IC

It is important to understand that all the experimental results have some range of uncertainty. For drawing valid conclusions from experimental results, the proper error analysis should be discussed. Therefore we performed different statistical analyses for the validation of our proposed macromodel. The proposed macromodel uses multiple regression to find the relationship between the power and statistical input variables.

A multiple regression model is fitted to find the exact relationship between the power for heterogeneous 3D IC and input variables and given in the equation below:

$$\begin{array}{c} Power_{Hetero_{3D}}=1.879+0.913.TD+0.155.SP-1.799.SV \end{array}$$ (23)

**Fig 9:**
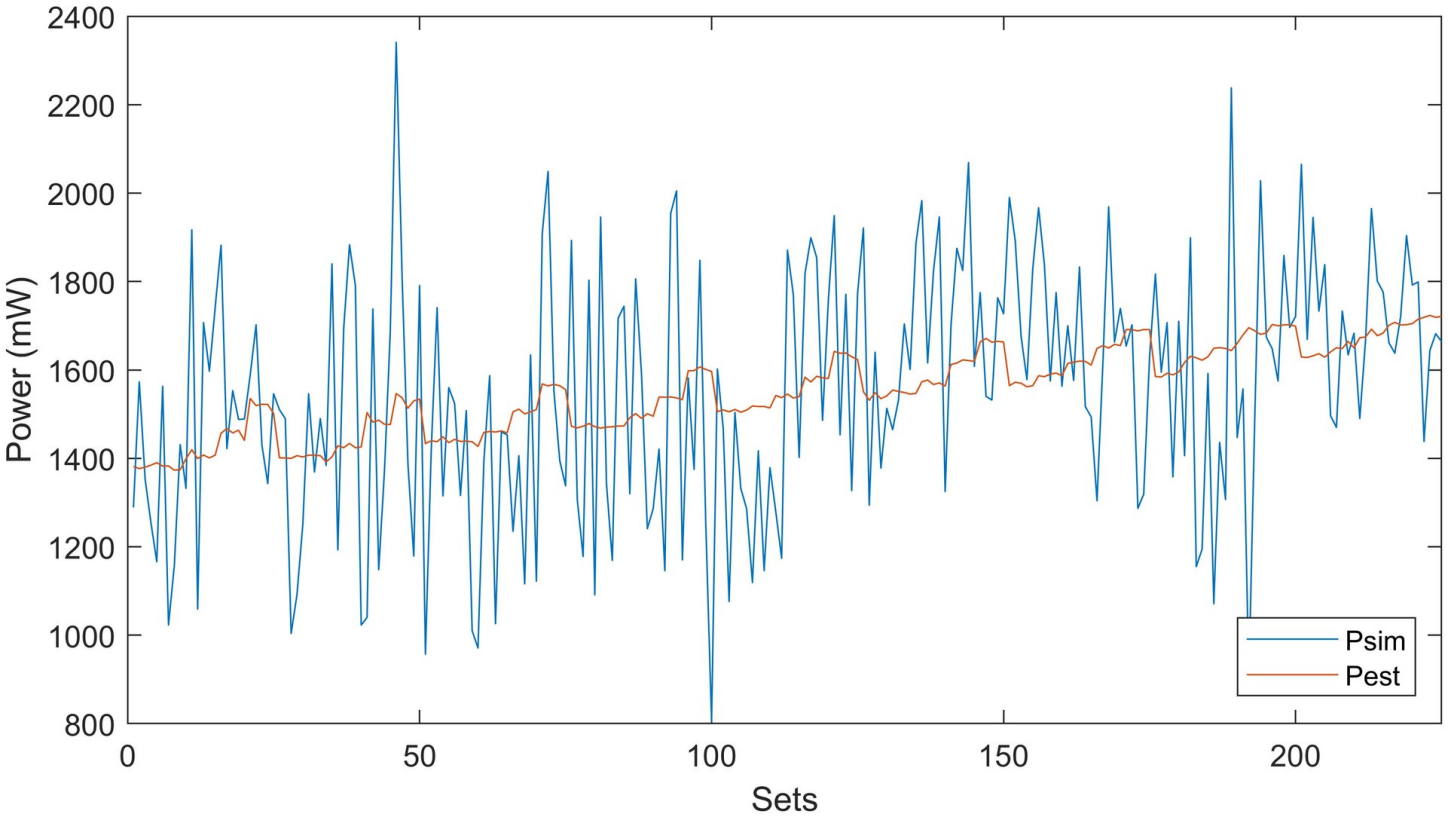
Correlation between simulated and estimated powers for heterogeneous 3D IC.

**Fig 10:**
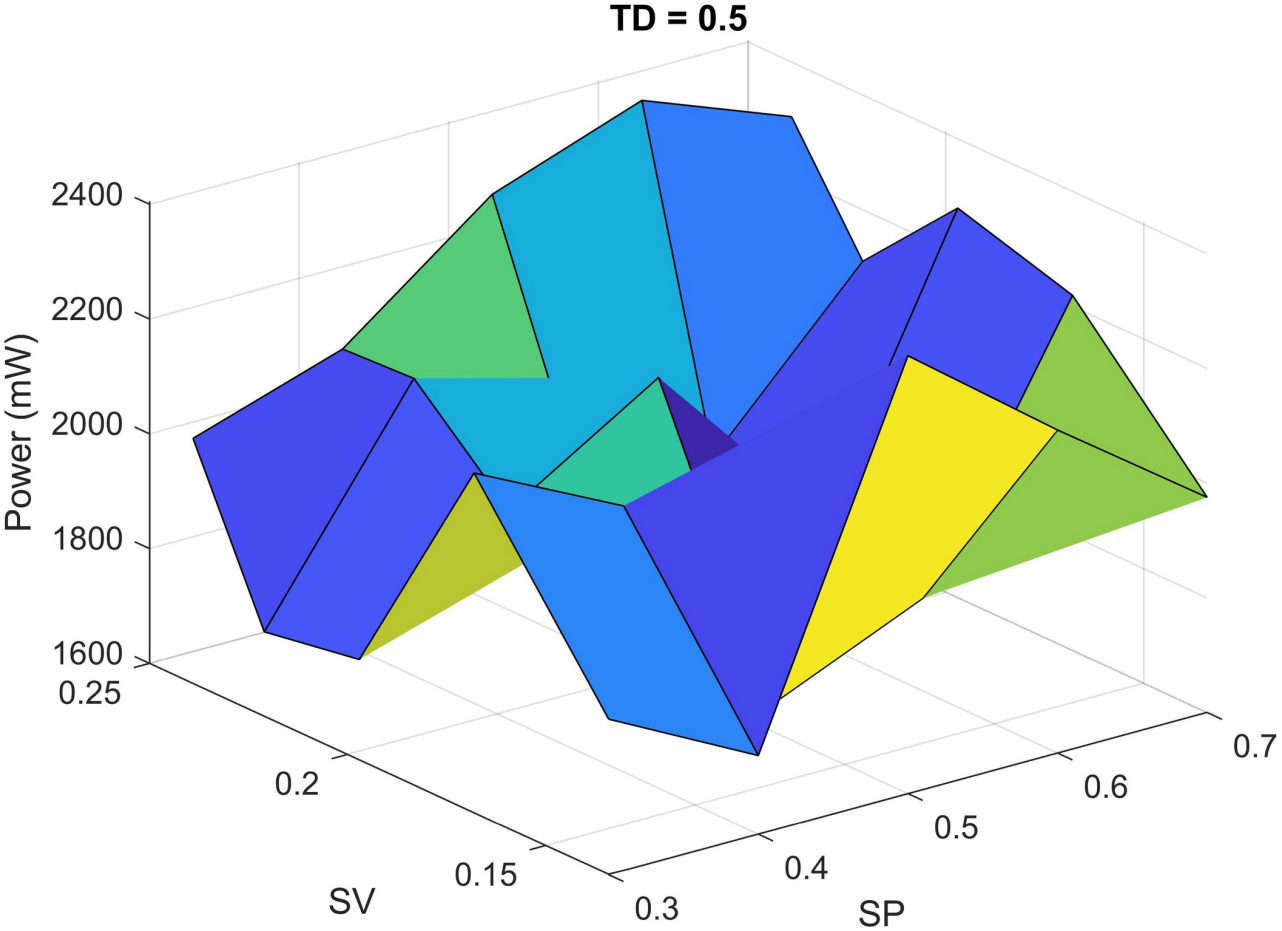
Power estimation for heterogeneous 3D IC by using proposed macromodel.

The statistical analysis for heterogeneous 3D IC is given in Table 7. Since all the P-values for input variables are less than 0.05, therefore there is a statistically significant relationship between the power and input variables at a 95% confidence interval. The correlation between the simulated and estimated powers is shown in Fig 9. Similarly, the power estimation for complete heterogeneous 3D IC at some specific values of parameters by using the proposed macromodel is shown in Fig 10. The *TD* is fixed at value of 0.5, and we vary the values of *SP* and *SV* in the span of [0 1] to make a 3D visualization of our propsoed mdoel. We can also choose any value of *TD* in the range of [0 1] to visualize the behavior of complete 3D IC.

**Table 7:** Statistical analysis of power for heterogeneous 3D IC.

Dependent Variable: Power		
Parameter	Standard Estimates	P-Value
Constant	1.879	0.000
TD	0.913	0.000
SP	0.155	0.046
SV	-1.799	0.005

### 4.9 Error analysis of heterogeneous 3D IC

A multiple regression model is fitted to find the exact relationship between the error and input variables and given in equation below:

$$\begin{array}{c} Error_{Hetero_{3D}}=-18.369-21.701.TD+2.585.SP+149.569.SV \end{array}$$ (24)

The statistical error analysis for Heterogeneous 3D IC is given in Table 8. Since the P-values for TD and SV are less than 0.05, therefore TD and SV are statistically significant parameters for error and input variables at 95% confidence interval. Regression is again performed with TD and SV and an updated equation is given as:

$$\begin{array}{c} Error_{Hetero_{3}D}=-15.635-21.716.TD+144.361.SV \end{array}$$ (25)

**Table 8:** Statistical error analysis for heterogeneous 3D IC.

Dependent Variable: Error		
Parameter	Standard Estimates	P-Value
Constant	-18.369	0.129
TD	-21.701	0.000
SP	2.585	0.669
SV	149.569	0.002

Detailed error analysis and power models are discussed in previous sections. The proposed model in (1) gives accurate results and also consumes less time compared to the previous model. By using BBO it needs just 109 s to generate 2250 sets of input patterns for each of the IP block/B-TSV/core/3D IC. On the other hand, our proposed model takes an average of 7.38 hours for Vivado simulations for each IP block/B-TSV/core/3d IC. This work is done by using multiple platforms by multitasking in order to save time. A comparison of the proposed macromodel with modified input variables with the previous macromodel given in the literature is given in Table 9. It is also evident from previous sections and discussions, that our proposed macromodel is efficient in terms of error accuracy. These results also show that our model is time-efficient as compared to previous ones. This is due to the introduction of a new statistical variable and also reduces the input variables and therefore the number of simulations required for each core, B-TSV and 3D IC is also reduced. Our model is more closer to the actual power estimation results obtained from the estimation tool. This gives a designer an early power estimate to redesign the power budget at the initial design stage.

**Table 9:** Comparison with previous model.

Sr. No:	Parameter	Proposed Model using BBO	Previous model using GA
1	Number of Simulations for each Core/B-TSV/Heterogeneous 3D IC	2250	11250
2	Number of Input Parameters	3	4
4	Percentage of Error of complete Heterogeneous 3D IC (%)	8.65	15.21

## 5 Conclusion & future work

It is evident from modern portable devices that low-power consumption is an important design concern that cannot be neglected. The main advantage of the proposed work is an early estimation of an IP-based 3D digital system before it will be manufactured. Early power estimation also gives a flexible solution for designers to modify their power budget, reliability and also reduces the turnaround time.

In this research, we have presented an improved statistical macro-modeling approach that estimates power through statistical characteristics of randomly generated input patterns by using the BBO. These input patterns propagate signals into IP-based 3D digital test system. In experiments, the test system is based on four 8 to 32- bits heterogeneous cores. The response of the power is monitored by applying the Monte Carlo Simulation technique. The entire power estimation method is performed in two major steps. First, the average power is estimated for an IP-based individual core. Second, the average power for B-TSV is estimated. Finally, the cores and B-TSVs are integrated together to construct a 3D system. Then the average power for complete test systems is estimated.

The experimental results of the statistical power macro-model are compared with the commercial EDA power simulator at the operating frequency of 100 MHz. The average percentage error of the test system is calculated as 8.65%. For the validation of these results, the statistical error analysis is additionally performed and reveals that our proposed macro-model is accurate in terms of percentage of error with a feasible amount of time. The proposed model can be used for low-power SoC commercial applications and it also saves time and budget by giving an early estimate of power consumption.

The error can be reduced by using the different power optimization techniques. One of the important sources of error is delay elements like glitches activities and jitters. Therefore, the Monte Carlo simulation approach with zero delay model with glitch model is a potential research area as a future direction for researchers.

A thermal model can be also proposed for thermal analysis of complete 3D IC for future recommendation. The error percentage can be further improved by adding models of glitches and jitters.

A hybrid model can be proposed for multi-cores by using the uniform/non-uniform physical model for B-TSV and the statistical model for multiple cores.

## Supporting information

S1 Fig Details of core-I. (TIF)

S2 Fig Details of core-IV. (TIF)

S1 File Fig 2 plot data. (XLSX)

S2 File Fig 4 plot data. (XLSX)

S3 File Fig 7 plot data. (XLSX)

S4 File Fig 8 plot data. (XLSX)

S5 File Fig 9 plot data. (XLSX)

S6 File Fig 10 plot data. (XLSX)

S7 File Track changes style file. (STY)
