# Supplementary figures and images for: Efficient power macromodeling approach for heterogeneously stacked 3d ICs using Bio-geography based optimization
Source: PLoS One. 2022 Feb 22;17(2):e0264181. doi: 10.1371/journal.pone.0264181 (PMC8863266; doi:10.1371/journal.pone.0264181)

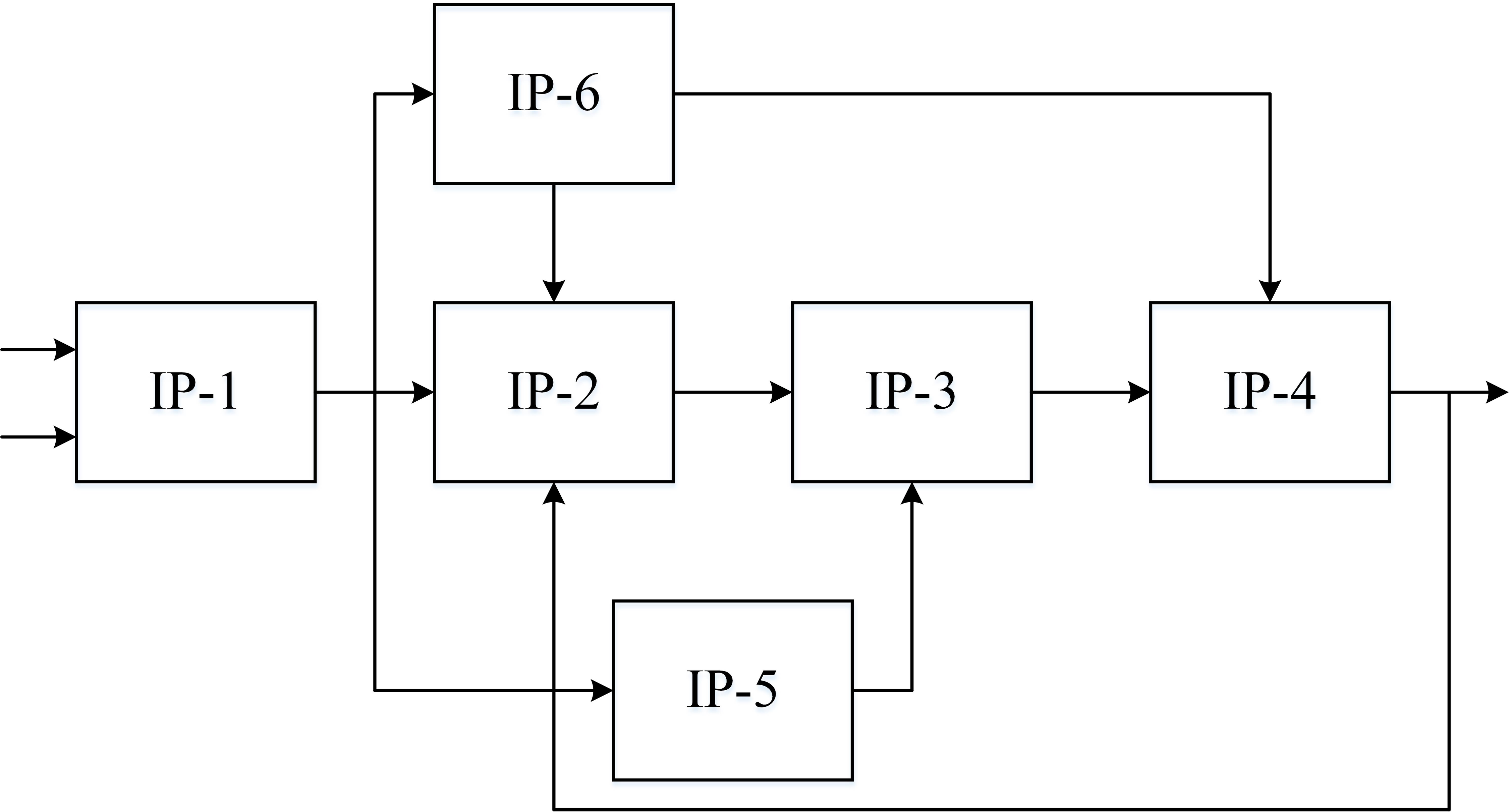

Supplement: S1 Fig — (TIF) [file pone.0264181.s001.tif]

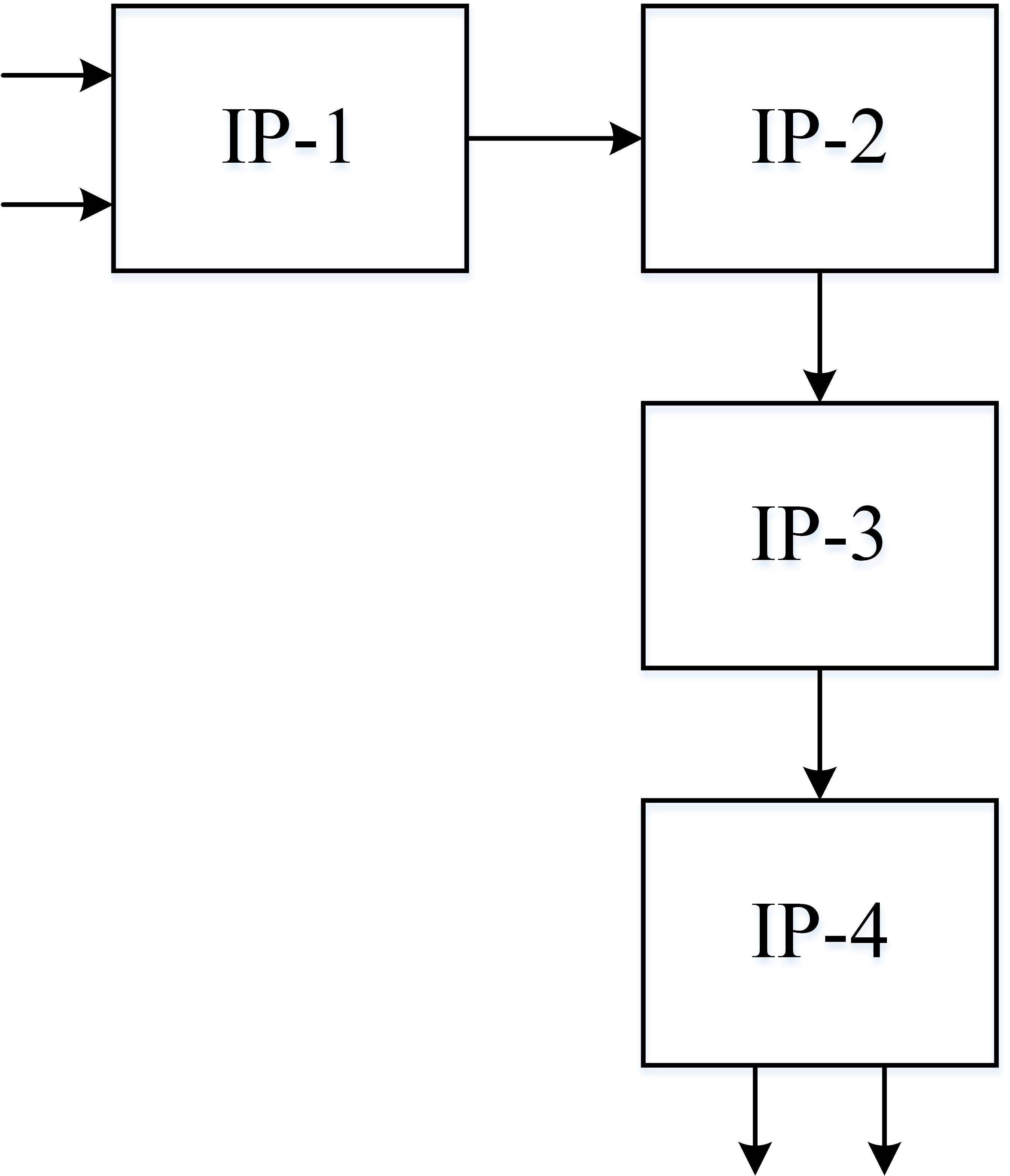

Supplement: S2 Fig — (TIF) [file pone.0264181.s002.tif]
